# Future Care Planning for and by Older Adults Living in the Community: A Scoping Review

**DOI:** 10.1177/01640275251348582

**Published:** 2025-06-08

**Authors:** Nathalie Bettina Neeser, Xin Feng, Wei Yang, Ruru Ping, Xue Bai, Tenzin Wangmo

**Affiliations:** 1 224633 Institute for Biomedical Ethics, University of Basel, Basel, Switzerland; 2 4616 King’s College London, London, UK; 3 12804 Hitotsubashi University, Tokyo, Japan; 4 26680 The Hong Kong Polytechnic University, Kowloon, Hong Kong SAR

**Keywords:** future care, care planning, long term care planning, elder care

## Abstract

In this scoping review, our aim was to compile empirical studies, outside of an institutionalized care context, that discuss future care planning (FCP) at old age for and by older adults living in the community. We define FCP as planning behaviours and decisions made in anticipation of a forthcoming period of life in which comprehensive care and support (i.e., financial, emotional, instrumental, and personal) become necessary due to aging and declining health. The search was conducted in eight databases and was limited to empirical papers published in English between 2000 and 2023. Only 12 articles fulfilled our inclusion criteria. The included studies were analysed narratively. The results were structured around six themes: (i) plans about who will provide care in the future when one no longer has the ability to do so themselves; (ii) plans about future housing and living arrangements; (iii) future financial planning; (iv) plans related to future health issues; (v) values communication as a means for planning; and (vi) the impact of formal care systems on FCP. Results highlight significant variability in how FCP is approached and call for further studies on this topic in light of not only demographic changes but also due to contextual differences in care provision and care expectations.

## Introduction

Advancements in science and medicine have substantially extended human life expectancy from 72.8 years in 2018 to 77.2 years in 2050 globally (United Nations, 2023), with older adults in some high-income nations enjoying life expectancies beyond their 80th birthdays. However, longer lifespans are often accompanied by an increase in physical and psychological conditions that hinder older adults’ ability to live independently. Worldwide, over 46% of older adults experience varying levels of disability (United Nations Population Fund, 2012). The combination of longer lifespans and a higher risk of disability means that older adults will increasingly rely on support from both family members and healthcare professionals. However, sociodemographic changes – such as declining fertility rates and the physical distance between parents and their children – are contributing to the shrinking pool of informal caregivers, making it difficult to meet the rising demand for care. Older adults often face difficult circumstances, with children who are preoccupied with their own families and careers or have moved away and are unable to offer hands-on support. As family caregiving becomes less available, formal health and social care services are also under increasing strain, facing rising demand and insufficient funding to meet the needs of the aging population, necessitating careful examination of future care planning (FCP). We define FCP as the process of considering future needs, engaging in planning behaviours, and making decisions in anticipation of care and support (i.e., financial, emotional, instrumental, and personal) that will become necessary due to aging and physical decline (Taubert and Duffy, 2024).

Such proactive care planning for old age has become increasingly important. Literature suggest that early planning is able to address potential future care needs and individuals can gain greater control over their care options, finances, and overall well-being (Broyles et al., 2015). For the aging population, the disciplines of medical and social sciences have predominantly focused their attention on end-of-life planning and decision making, advance directives, palliative care, and hospice care (Carr & Luth, 2017; Dickinson et al., 2013; Hickman et al., 2023; Mullick et al., 2013). It is evident that the topic of advanced care planning (ACP) has received much attention (Frechman et al., 2020; Hopkins et al., 2020; Jimenez et al., 2018; Sharp et al., 2013). Unfortunately, the focus has largely remained on medical decision making at the very end of older adult’s lives and for situations where the person is incapable of making an informed decision. This underlines the need to study care planning at an earlier stage, from a more proactive perspective and not limited to the very end of the older adults’ lives or when they have already become dependent on others, for example when receiving institutional care.

In reviewing the current literature, the majority of studies focus more broadly on coping with frailty, whether anticipated or present, rather than specifically on planning for an optimal future life and care situation at old age (Corry et al., 2021; Turjamaa et al., 2015). Others focus on care goals of older people (Clair et al., 2021; Giosa et al., 2022; Kuluski et al., 2013), their personal expectations and preferences for care (Bai, 2018, 2019; Bai et al., 2020; Malhotra et al., 2012; Pinquart & Sörensen, 2002a) and the experiences of those receiving care in institutional settings (Sörensen et al., 2008; Southerland et al., 2016). In addition, frameworks and scales have been proposed to understand and measure how older adults and their formal and informal caregivers might be involved in care decisions. Sörensen and Zarit (1996), for example, proposed a framework to understand the planning of older adults’ future and concluded that, although the thinking about and talking of future care needs often come together, that this does not necessarily mean that the older adults in question formulate any concrete future care plans. Other scholars have developed, tested, or validated scales for future care planning (Allen et al., 2018; Bai et al., 2022; Sörensen et al., 2017; Sörensen and Pinquart, 2001; Wolff et al., 2021).

Our aim in this scoping review was, to compile empirical studies that discuss FCP for older adults who are not (yet) receiving institutionalized care. That is, we aim to study whether and how FCP was carried out for and by older persons living in the community, who were generally healthy and not in need of intensive care. In doing so, we mapped the different processes and aspects involved in FCP for older adults and chose to strongly centre our review on the planning and decision making aspects involved when it comes to future care. As such, it was important to, for example, exclude studies focusing on goals of care, as they do not pointedly focus on more comprehensive planning processes. Due to the general lack of studies when it comes to FCP related studies, and the broad nature of our aim, we chose to implement the methodology of a scoping review, instead of the one of a systematic review (Peters et al., 2015).

## Research Design

We used the framework of Arksey and O’Malley (2005) to identify and select eligible articles, and to collate and summarize the results. We also followed the PRISMA Extension for Scoping Reviews (PRISMA-ScR) and Peters and colleagues’ (2015) guidance for conducting systematic scoping reviews (Peters et al., 2015) to ensure the quality of the scoping review and to allow it to be reproducible (Tricco et al., 2018).

### Criteria and Rationales for Inclusion and Exclusion

For studies to be included in the review, we set four specific inclusion criteria: (i) English language empirical studies published in peer-reviewed journals between January 1, 2000 and December 31, 2023; (ii) the study participants were older adults (60 years and older, or the mean age of the sample (where provided) was at least 60 years) and/or adult children or other informal carers discussing future care planning at older age when caregiving becomes necessary; (iii) older adults in the study must not be receiving institutional care (i.e., living in assisted living, nursing home or old age home); and (iv) old age FCP must be the key aim of the included paper or data relevant for this topic is retrievable from the results, such as, housing, financial, activities of daily living (ADL) support planning, long term care planning as well as behavioural actions towards future care. This meant that we excluded: (i) theoretical studies, dissertations, theses, reviews, book chapters, conference proceedings and publications in other languages than English; (ii) studies where the older adults were below 60 years old (or where the mean age was younger than 60 years); (iii) studies where an older adult was receiving institutional care (i.e., medical care, nursing care); and (iv) studies that were related to advanced health care planning such as end-of-life decision making, ACP, palliative care, care goals, or care expectations.

As the study aims to evaluate care planning of and/or for older adults, our goal was to gather all the existing empirical evidence from peer-reviewed manuscripts, as they underline higher quality data than grey literature or those published in books or conference proceedings, which may not be peer-reviewed. We further chose the rationale of excluding any studies carried out with older adults already receiving care in institutionalized settings because for them, there is limited further plans to be made as they are already receiving institutionalized care and such plans are likely to be medical in nature often surrounding ACP, for which ample research exists (Brinkman-Stoppelenburg et al., 2014; Frechman et al., 2020; Hopkins et al., 2020; Jimenez et al., 2018; Pimsen et al., 2022; Sharp et al., 2013).

### Search Strategy and Terms

The protocol for this scoping review was registered in Open Science Framework (OSF) and can be accessed online (Wangmo et al., 2024). Our search strategy aimed to capture studies from disciplines such as Medicine, Nursing, Sociology, Psychology, Gerontology, and multidisciplinary databases. The databases included: (i) PubMed incl. Medline; (ii) CINAHL; (iii) PsycInfo; (iv) SocINDEX; (v) Web of Science; (vi) Scopus; (vii) Embase; and (viii) Google Scholar. To fit the specific aim of the review, we designed the search strategy based on a Population I, Population II, Context (PPC) structure (see Table 1), which is an adaptation of the traditional population intervention comparison outcome (PICO) scheme. Within the PPC components, all terms were linked to each other by Boolean ORs, while the components were linked with Boolean ANDs (See Tables 1 and 2). The search strategy and terms were developed and agreed upon by all authors and the final search was run on January 29, 2024 and not re-run prior to the final analysis. Covidence was used for citation management.

**Table 1.** Search Strategy and Terms.

Structure^a^	Definition	Search terms
Population I	Older adults	older adult* OR ag*ng OR elder* OR aged person* OR older person* OR elder* people OR older parent* OR older people OR elder* person* OR senior* OR geriatr* OR gerontolo*
Population II	Family members of older adults	informal caregiv* OR unpaid caregiv* OR famil* caregiv* OR caregiv* OR carer* OR informal carer* OR unpaid carer* OR famil* carer* OR adult child* OR adult daughter* OR adult son*
Context	Future Care Planning for old age	care* plan* OR care* prepar* OR care* discussion* OR social care plan* OR long-term care plan OR anticipatory care planning

^a^Population I, Population II, and Context were linked to each other with Boolean ANDs.

**Table 2.** Full Search Strategy for Embase.

Population I, Population II & Context	('older adult'/exp OR ‘older adult' OR ‘older adults'/exp OR ‘older adults' OR ‘aging'/exp OR ‘aging' OR ‘aged'/exp OR ‘aged' OR ‘older people'/exp OR ‘older people' OR ‘senior resident'/exp OR ‘senior resident' OR ‘geriatric'/exp OR ‘geriatric' OR ‘geriatrics'/exp OR ‘geriatrics' OR ‘gerontology'/exp OR ‘gerontology' OR ‘older adult*':ab,ti OR ‘ag*ng':ab,ti OR ‘elder*':ab,ti OR ‘aged person*':ab,ti OR ‘older person*':ab,ti OR ‘elder* people':ab,ti OR ‘older parent*':ab,ti OR ‘older people':ab,ti OR ‘elder* person*':ab,ti OR ‘senior*':ab,ti OR ‘geriatr*':ab,ti OR ‘gerontolo*':ab,ti) AND ('informal caregiver'/exp OR ‘informal caregiver' OR ‘caregiver'/exp OR ‘caregiver' OR ‘family caregiving'/exp OR ‘family caregiving' OR ‘adult child'/exp OR ‘adult child' OR ‘informal caregiv*':ab,ti OR ‘unpaid caregiv*':ab,ti OR ‘famil* caregiv*':ab,ti OR ‘caregiv*':ab,ti OR ‘carer*':ab,ti OR ‘informal carer*':ab,ti OR ‘unpaid carer*':ab,ti OR ‘famil* carer*':ab,ti OR ‘adult child*':ab,ti OR ‘adult daughter*':ab,ti OR ‘adult son*':ab,ti) AND ('care* plan*':ab,ti OR ‘care* prepar*':ab,ti OR ‘care* discussion*':ab,ti OR ‘social care plan*':ab,ti OR ‘long-term care plan':ab,ti OR ‘anticipatory care planning':ab,ti)

With the additionally applied filters: “time” (2000–2023), “human”, “language” (English) and “publication type” (Article, Article, in press).

### Study Selection and Screening Process

The screening process of all remaining articles after automatic and manual duplicate removal began with the evaluation of the titles and abstracts. To do so, the titles and abstracts of articles were screened independently by two researchers (XF and RP) against the inclusion and exclusion criteria. In case of disagreement, two other reviewers (NN and WY) decided on inclusion or exclusion. All full-texts of articles included from the first screening process were then read and reviewed. This second screening process included two reviewers (NN and XF), who again assessed the eligibility of these studies and extracted the data relevant for this scoping review. To do so, a data charting form was developed by TW, WY and XB, based on their knowledge of the field. It included information on the included studies such as: (i) the goal of the paper; (ii) participant information, such as the sample size and the age of study participants; (iii) the methodology used in the study; (iv) study findings relevant to FCP; (v) the scope and nature of FCP, for example housing, financial, social, etc.; (vi) limitations of the topic identified in the study; (vii) limitations of the study methodology; and (viii) recommendations for future research. The extraction form was pilot tested with a few included studies to ensure that all necessary data will be gathered. To ensure completeness and a sound extraction of the data in question, the extracted data of one reviewer was checked for completeness by a second reviewer. We did not engage in an assessment of methodological quality of the included studies, as this is not a necessity for scoping reviews (Tricco et al., 2018).

**Figure 1.**
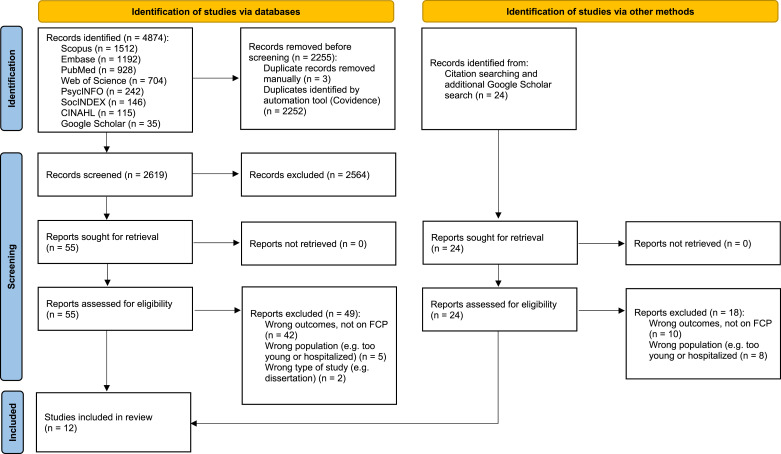
PRISMA flow diagram. *From:* Page MJ, McKenzie JE, Bossuyt PM, Boutron I, Hoffmann TC, Mulrow CD, et al. The PRISMA 2020 statement: an updated guideline for reporting systematic reviews. BMJ 2021;372:n71. doi: 10.1136/bmj.n71.

All screening processes were carried out in Covidence, which allowed clear management of the data and ensured that all reviewers were able to see the work in progress and resolve discrepancies when needed. A PRISMA flow diagram was created to present the workflow of this scoping review (Figure 1). For data analysis, Microsoft Excel was used to carry out analyses of the data. We used narrative synthesis of review data to analyse and make sense of all data charted in this scoping review (Popay et al., 2006). Narrative synthesis is a useful tool for analysis, as it is often a challenge to find common denominators besides the research question when engaging in a review, especially when studies were conducted in different socio-cultural contexts, publications are of different quality and different methodologies were employed (Popay et al., 2006). Narrative synthesis is a form of storytelling and predominantly uses text and words to summarize and explain the findings of data stemming from different methodologies, which is the case when a data corpus is composed of published studies. The charting of themes developed from the analyses were iteratively discussed among the co-authors and changes were made when necessary.

## Results

From the eight searched databases, a total of 4874 entries were identified. After automatically and manually removing duplicates, 2619 studies remained, which were independently assessed by two reviewers resulting in the removal of 2564 studies. Thereafter 55 studies remained for the full-text assessment, to which, 24 additional studies were added based on citation searching and a Google Scholar search for publications of specific scholars (namely Silvia Sörensen and Martin Pinquart), whose studies were repeatedly cited in the included papers. During the full text screening, further 67 studies were excluded (see Figure 1). Examples of studies that we initially assessed to be included but were excluded during the full text screening were, for example, studies focusing on discussions about decisions made retrospectively (Adekpedjou et al., 2018; Chiang-Hanisko, 2010); studies with participants already receiving care in institutional settings (Sörensen et al., 2008; Southerland et al., 2016), studies where the aims were solely to develop or test future care planning scales (Sörensen et al., 2017; Sörensen and Pinquart, 2001), studies where authors focused on care goals (Clair et al., 2021; Giosa et al., 2022), or studies with a focus on personal care expectations or preferences (Bai, 2018, 2019; Bai et al., 2020; Pinquart & Sörensen, 2002a).

Therefore, this review includes 12 studies that specifically discussed FCP as defined in this study, spanning a period from 2000 to 2023. Geographically, three were from Asia (China and Thailand), six from the United States (US), and three were comparing samples from two or more countries (US, Canada and/or Germany). Eight studies squarely focused on FCP, while in the remaining four studies FCP was one of the topics studied. In nine of the 12 included studies, all study participants were older adults, and in three there was a mix of older adults and informal caregivers. None of the studies solely focused on the perspectives of informal caregivers (Table 3). Six studies used quantitative methods of surveys or secondary analysis of existing datasets and six studies used interview methods.

**Table 3.** Study Characteristics.

Last Name First Author	Year of Publication	Title of Publication	Country in Which the Study was Conducted	Study Approach	Study Sample
Sörensen	2000	Preparation for future care needs: Styles of preparation used by older Eastern German, United States, and Canadian women	East Germany, United States & Canada	Qualitative interviews	*N* = 45 older persons, ranging in age from 65 to 86 years
Roberto	2001	Older adults’ preferences for future care: Formal plans and familial support	United States	Qualitative interviews	*N* = 45 older persons, ranging in age from 56 to 88 (mean age = 71.1 years)
Pinquart	2002	Factors that promote and prevent preparation for future care needs: Perceptions of older Canadian, German and U.S. women	Germany, United States & Canada	Qualitative interviews	*N* = 45 older persons, ranging in age from 65 to 86 years (mean age = 76.2 years)
Pinquart	2003	National and regional differences in preparation for future care needs: A comparison of the United States and Germany	United States & Germany	Quantitative survey	*N* = 1172 older persons, ranging in age from 65 to 93 years
Delgadillo	2004	An Exploratory study of preparation for future care among older Latinos in Utah	United States	Quantitative survey	*N* = 350 older persons (62 Hispanic older persons (mean age = 64 years) and 288 non-Hispanic older persons (mean age = 74.4 years)
Kim	2014	Home modifications by older adults and their informal caregivers	United States	Quantitative analysis of secondary data	*N* = 737 older persons; *N* = 737 informal caregivers
Girling	2014	Older women discuss planning for future care needs: An explanatory framework	United States	Qualitative interviews	*N* = 185 older women, ranging in age from 64 to 98 years
Pothisiri	2018	Preparations for old age and well-being in later life in Thailand: Gender matters?	Thailand	Quantitative analysis of secondary data	*N* = 10’235 older persons (60 years and older)
Orsulic-Jeras	2019	The SHARE program for dementia: Implementation of an early-stage dyadic care-planning intervention	United States	Qualitative interviews	*N* = 40 older persons (mean age = 74.2 years); *N* = 40 informal caregivers (mean age = 61.3 years)
Chen	2020	Life restoration and future care preparation among older parents in Shanghai who have lost their only adult child	China	Qualitative interviews	*N* = 24 older persons, ranging in age from 61 to 85 years (mean age = 69 years)
Kozlov	2021	The feasibility and acceptability of an intergenerational, web-based intervention to enhance later-life family care planning	United States	Quantitative survey	*N* = 49 older parents, ranging in age from 65 to 83 (mean age = 72.1); *N* = 93 adult children, ranging in age from 25 to 65 (mean age = 45.7)
Bai	2022	Adaptation and validation of the preparation for future care needs scale for Chinese older adults in Hong Kong	China	Quantitative survey	*N* = 862 older persons aged 60 years or older (mean age = 72.5 years)

**Figure 2.**
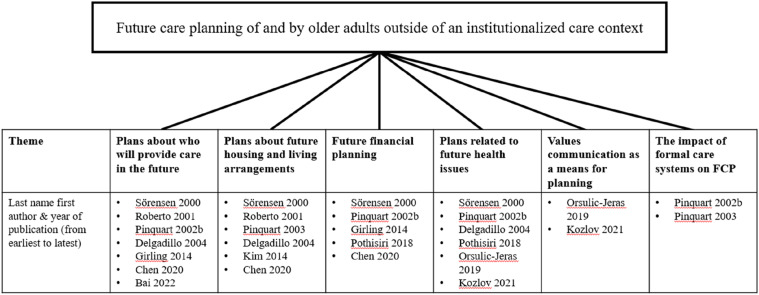
Thematic overview.

By engaging in narrative synthesis and through iterative discussions, we structured our results around six distinct thematic aspects in connection to FCP: (i) plans about who will provide care in the future (when one no longer has the ability to do so themselves); (ii) plans about future housing and living arrangements; (iii) future financial planning; (iv) plans related to health issues; (v) values communication as a means for planning; and (vi) the impact of formal care systems on FCP (see Figure 2 and Table 4).

**Table 4.** Summary of Key Study Results Related to Future Care Planning.

Study	Results
Sörensen and Pinquart (2000)	• Preference for family care, may use home care or nursing home services for short- or long-term care needs. • Reluctance to plan due to anxiety about nursing homes, lack of alternatives, and high costs. • Limited financial resources lead to reliance on nursing homes, as the only option. • Proactive participants were aware of their current health status and their future care needs.
Roberto et al. (2001)	• Care expectations from family and from formal services. • Geographical distance did not disqualify children as future caregivers. • Preferences are shaped by past, present, and anticipated future family relationships, but the quality of the relationships does not always predict these preferences. • Those with sufficient financial resources had made formal plans (e.g. long-term care insurance, retirement community homes, nursing home provisions).
Pinquart and Sörensen (2002b)	• Avoidance of planning due to the belief that doctors or adult children would make better plans for them than themselves. • Did not expect to need care in the future, avoided considering future care risks, and avoided future planning due to insufficient financial resources. • East Germans were more likely to report not having plans due to financial resource constraints than Canadians and Americans. • To cope with uncertainty, older women often reported having no or only general plans, which can be adjusted or changed.
Pinquart et al. (2003)	• Germans favoured home health care plans, while Americans preferred home remodelling. • West Germans were more likely than East Germans to plan for assisted living, home remodelling, and the use of home health care or family support. • Differences between the US and Germany due to beliefs about healthcare system stability (e.g., Germany’s long-term care insurance reduces the need for individual planning).
Delgadillo et al., (2004)	• Latinos preferred family care compared to Anglo-Americans and were also less likely than Anglo-Americans to have concrete plans. • Negative attitudes to assisted living or nursing home services and opposition to move to an institutionalized setting.
Kim et al. (2014)	• Home modifications are essential for aging in place. • Younger, cohabiting caregivers are more likely to modify homes.
Girling & Morgan (2014)	• Different planning strategies: Autonomous planners, passive planners who relied on others, thinkers who did not take any action, optimists who saw no need, and avoiders. • The avoiders avoided because of low self-perceived vulnerability, financial constraints, lack of information, and fear of burdening others. • Financial barriers impact African American women less due to their care preferences from family members, while European American women viewed family members as an alternative as future care providers due to limited finances.
Pothisiri and Quashie, (2018)	• No gender differences in financial satisfaction post-retirement in the study population. • Older men were slightly more likely to plan for the future, were more educated and had higher financial stability than older women. • Older women had the tendency to receive money from children, while older men provided money to their children. • Women were less likely than men to engage in health and residential planning, and those who did had better post-retirement health.
Orsulic-Jeras et al. (2019)	• Care dyads felt better prepared and more confident in making care decisions after participating in the FCP intervention. • Care partners felt less burdened after aligning care decisions with the older person’s values.
Chen et al. (2020)	• Community care was perceived as patronising, leading to reluctance to discuss non-family care options. • Most participants made no plans for future living arrangements, expecting government-provided nursing home care. • Only one study participant had made a plan by moving to a smaller apartment after selling their home. • Concerns about potential financial difficulties as in China adult children traditionally support their parents, and the study population had lost their only adult child.
Kozlov et al. (2021)	• Agreements between the older person and family members across five areas related to the older person’s quality of life, housing, legal documents regarding ACP, end-of-life care, and communication regarding FCP. • Web-based tools enhance communication between family members and help older people understand their FCP preferences.
Bai et al. (2022)	• Validation of the Chinese version of two short forms of the PFCN scale to align with local socio-cultural norms. • Care planning is influenced by cultural values prioritising family care. Limited formal care options are available.

### Plans About Who Will Provide Care in the Future

Seven studies provided information on plans related to who will provide the necessary care to an older adult in need. The earliest study on the topic included in our review was carried out in Germany, the US and Canada (Sörensen and Pinquart, 2000). In their study, the authors characterized the 45 female participants with chronic care needs into four styles when it comes to planning. The first planning style included those who felt that planning may be futile and outside forces such as doctors, agencies and family members would make decisions for them when needs eventually arise. The second group of planners included those who reported having ambivalent relationships with their family members and thus could not count on them in ensuring their well-being when in need of care. Thus, they made plans in light of a lack of availability of family members. In a similar but different vein, the third group of planners were those who did not wish to burden their family members and thus made plans to receive home care services or enter nursing homes. The fourth and final group included those participants who planned to meet their chronic care needs by making financial arrangements that would secure the support they need or made agreements with family members who would take care of their needs. Not being a burden on family members was important to study participants.

Solely in the US context, Roberto and colleagues (2001) found that half of their older study participants expected that their *family members* (children or grandchildren) would provide care for them when needed, and the other half of the participants expected to receive care from *formal sources*. Authors stated that participant’s preferences, about who is to take care of them, were shaped by their past, present and anticipated future with their family members. However, authors also found that the quality of the relationship does not necessarily predict the older adult’s FCP preferences. In addition to family members, in Pinquart and Sörensen’s study (2002b), a small group of participants were lacking in FCP and rationalized that others will make better plans than them, such as their *doctors* in the case of long term care needs (i.e., placement in a nursing home), or their adult children who will plan for all care needs. This finding was similar to their previous study (Sörensen and Pinquart, 2000). They also found that planning for the future was viewed as a way of reducing the burden on future caregivers and of preventing potential conflicts (Pinquart & Sörensen, 2002b). However, it is also noteworthy that some participants did not expect to need care in the future at all. Authors state that these participants avoided thinking about future care risks, and therefore, risked that future care needs might not be met.

Indeed, not all older adults would rely on “others” to care for them. Girling and Morgan (2014) studied FCP with a sample of 185 older women, and categorized the women into five planning groups. One group of participants were characterized as “Autonomous Care Planners” who had purchased long term care insurance and made contracts guaranteeing that continued care in times of need was set in place. However, another group of participants were characterized as the “Externally Reliant Planners” who share similarities with the studies presented above (Pinquart & Sörensen, 2002b; Roberto et al., 2001). That is, these women relied on others (family members or friends, and organizations that they belonged to) to take care of their future long-term care needs. From Girling and Morgan’s (2014) study, we hence learn that women belonging to the groups of the autonomous care planners and the externally reliant planners, did plan for their futures to some degree. However, women belonging to the aware of preference, wishful thinkers, and the avoiders did not plan for their future needs in the present. This study, by providing a categorization of planning types, nicely underlines the variability in FCP from those who do not wish to make FCP to those who make more concrete plans about their future self.

In Chen and colleagues’ study (2020), older parents living in China were able to meet their own needs at the time of the interview. Although they had previously lost their only adult child, they refused to discuss any future caregiving situation other than *family caregiving,* underlining the value of children in the context of future care planning. Their refusal was based on the belief that community care was patronizing and would expose their vulnerable situation of not having a younger family caregiver. Additionally, participants were reluctant to engage with their potential care needs, since preparing reminded them of their loss. Different from Chen and colleagues’ study, the participants in Roberto and colleagues’ (2001) study noted no stigma in being cared for through formal care services such as in-home services and the placement in a nursing home. Another study with Chinese older adults reported their lack of engagement with care planning underlining the role of sociocultural factors, such as the reduced availability of formal care options as well as negative perceptions associated with institutional care, and traditional values of family care (Bai et al., 2022). Hence the authors concluded that future care planning among older adults in Hong Kong was influenced by cultural values placed on family care and underscored by a high reliance on family members and limited formal care options available to these older adults. Similarly, in a quantitative study, Delgadillo and colleagues (2004) revealed cultural differences in who would be the caregivers in case of need. Although Latinos were generally less likely than Anglo American study participants to report having concrete plans, they were significantly more likely to prefer family care in comparison to Anglo-American study participants.

It becomes clear, that both the cultural context in which one lives, as well as the availability and the quality of relationships with family members shape the plans about who will provide care when someone no longer has the ability to do so themselves. Additionally, it appears that the personality of older adults and the familial context in which they live shape their planning for the future, as several authors pointed out observing and analysing dissimilar planning behaviours within a particular group of people (Girling & Morgan, 2014; Sörensen and Pinquart, 2000).

### Plans About Future Housing and Living Arrangements

Six studies included discussions surrounding plans made for older adults’ living situations when they would no longer be able to live independently. Only one study had a focus on home modification (Kim et al., 2014), highlighting sociodemographic characteristics that predict home modifiers. The study focused on non-Hispanic Caucasian caregiving dyads in rural areas of the US, where the informal caregivers were younger than the care recipient and were living with and taking care of them. Besides home modifications, some older adults decided to move to new places to better receive future care. However, it is noteworthy that authors report home modification to be an important contribution to multidisciplinary care and for older adults to potentially age in place. In Chen and colleagues’ (2020) study, only one older Chinese study participant (of a total of 24 participants) had planned to move to a smaller apartment after the sale of his home which would allow him to secure funds for his future living expenses. Other study participants expected the government to manage their living arrangements in old age by placing them in nursing homes.

In the study of Roberto and colleagues (2001), nine persons (20% of the sample) with sufficient financial resources made specific arrangements for their future care needs and their future living situation. This included, for example, having long-term health insurance, having a place in a long term care (LTC) retirement community, and ensuring that their nursing home costs were covered. Another 15 participants (33% of the sample) anticipated making plans such as using home-care services and entering nursing homes when they would be unable to live independently. Pinquart and colleagues (2003) found that in the German context, regional differences persisted in the planning for future living arrangements. They revealed that for long term care, West Germans were more likely to have plans to move into an assisted living facility, more likely to remodel their current living space, and more likely to use home health care services or depend on family members than East Germans.

Sörensen and Pinquart (2000) revealed that participants characterized by an avoidance of planning had anxiety associated with going to a nursing home, either due to existing social attitudes towards this form of old age care (in the US) or due to their inability to afford nursing home care (in East Germany) that made such planning fruitless. The lack of enthusiasm to move to a nursing home at old age was evident in the study by Delgadillo and colleagues (2004), where about 80% of the respondents (*N* = 350) answered negatively to questions asking them about plans to live at an assisted living facility or a nursing home. However, in Sörensen and Pinquart’s study (2000), those participants (both in the US and West Germany) who were financially capable had made plans for their old age living arrangements, including home care and co-living as long as possible, with the plan of entering a nursing home when aging at home was no longer an option.

From the above, we find that when it comes to planning about future housing and living arrangements, the included studies underline that there is a strong financial ability aspect linked to it. That is, it is mostly those who can financially afford to think about and act on living arrangement plans, who are the ones doing so. Plans about housing not only relates to older adults’ financial circumstances, but also to the specific health and long-term care systems around them. In certain cultural context, living in residential or care facilities may also be linked to stigma.

### Future Financial Planning

Five studies addressed the topic of financial worries or security for FCP, mainly discussing the absence or presence of financial or social resources to ensure the ability to financially take care of future care needs. In the socio-cultural context of China, children tend to provide financial care to their parents, but in Chen and colleagues’ (2020) study, the older Chinese parents in question had lost their only child. This meant that they had no one to financially and socially rely on. They thus expressed their concerns about anticipated financial hardships as they would not be able to afford nursing home costs in the future. The data from Thailand on older adults (*N* = 10,235) planning for their future care needs reported that older men had higher financial stability than older women, with older women more likely to receive money from their children and older men more likely to provide money to their children (Pothisiri and Quashie, 2018). The study also noted that older men were more likely than older women to invest in their financial planning (63% vs. 56%) and those (both women and men) who made financial preparations for retirement displayed greater financial satisfaction post-retirement.

Also, Girling and Morgan (2014) reported that financial constraint was a barrier to making future care plans for the older women in their sample. Their study showed that to those who were financially insecure, the high cost of long-term care insurance was a deterrent to making any plans. However, some may not have made plans for long-term care because such facilities were deemed “the bad place”, and they expected that family members would take care of them in the future. The authors noted that African American women were more likely to not mention financial insecurity when discussing not having a plan, but mentioned that they had other care goals. Different from this group, European and American women reported that their family members were their care alternatives in light of lacking financial means.

Sörensen and Pinquart (2000) also underlined the relationship between having limited financial and social resources and the ability to plan. In the case of the participants from the US, not having financial resources combined with the only option available to them being in nursing home care, the elders were reluctant to make any plans and thus avoided doing so. The study also revealed that some plans made for the future (e.g., nursing home) by East Germans were not an option after the reunification of Germany as the cost of nursing homes had increased, making their plans infeasible and leaving them to become a burden to their family. Those who had limited financial resources but were not financially insecure were only able to make short-term plans. And those who had financial stability were the ones who were more likely to make long-term plans, focusing on how care could be provided at the place of their choosing or that they would be able to live out their life in a nursing home facility, in comparison to those who were not financially secure. Similarly, in Pinquart and Sörensen’s (2002b) study, participants reported not making plans for the future since they did not have enough financial resources to make reliable plans, with East Germans more often reporting that they did not plan for the future in light of lacking resources than participants from Canada and the US.

When it comes to financial planning, all seems to come down to the question, whether someone has the resources to even *think* about engaging in financial planning or not. However, not only the resources in relation to someone’s finances play a role when it comes to questions related to financial planning, but also one’s resources in terms of relationships to family members, specifically to offspring. Finally, included studies also show that there is a gender component to financial planning, pointing out that men had both more financial resources and were more likely to engage in financial planning in comparison to women (Pothisiri and Quashie, 2018).

### Plans Related to Future Health Issues

Six studies included information on future health planning. In Kozlov and colleagues’ study (2021), authors outlined the overall agreement among family members across health planning areas such as parent’s valuations of quality of life in different health scenarios, whether parents had completed legal documents to assure their ACP, and parent’s end-of-life care preferences. Also, in a dyadic intervention study of 40 older adults and their caregivers, Orsulic-Jeras and colleagues (2019), examined care values and preferences of older adults and their caregivers. They found that post study intervention, the majority of care dyads strongly agreed that they understood the illness better, felt more control over the care situation, felt better prepared for what lies ahead, and were more confident making care decisions. Sörensen and Pinquart (2000) found that those participants who planned for their future were financially able to do so and were aware of their future health care needs. These participants were aware of their frailty, were ill, and thus took charge of the needed decisions. Using the data from the same sample, another paper from this project showed that planning for future healthcare needs was also a way to cope with health challenges faced in the present (Pinquart & Sörensen, 2002b). By demographic characteristics, Pothisiri and Quashie’s (2018) study reported that women were less likely than men to engage in preparations related to their health care needs and their future living situation. However, when older Thai women did engage in health preparations, the women in question had higher odds of experiencing good health during their post-retirement years. Finally, with respect to racial differences, Delgadillo and colleagues (2004) found that more than four-fifth of their Latino study participants made no short term or long term health care plans, whereas this was the case for two-fifths of the Caucasian respondents (the racial difference was statistically significant).

Therefore, there are benefits in actively engaging in planning for future health issues. Not only did the importance of understanding an illness and its likely course play a role for participants in the included studies, but it was also shown, that open communication with family members is essential.

### Values Communication as a Means for Planning

A topic, which was of utmost importance and specifically highlighted in two studies, was later-life family communication. Kozlov and colleagues (2021) showed in their study that FCP can be improved using web-based tools that allow enhanced communication between family members to learn about the care preferences of older adults. In light of geographical distance between older adults and their children, as well as a lack of resources to come together to have such discussions in-person, the availability of online-tools stands to provide easier means to ensure that care planning for older adults is carried out in such a way that their values and wishes are incorporated. Furthermore, Orsulic-Jeras and collegues (2019) found that early discussion of the older adult’s values and preferences when it comes to future care, allows families to approach future care plans in a systematic way. The study found that the care-dyad’s understanding of the importance of values for the older adult helped the caregivers to explore possibilities to address future care needs that align with those values, which also reduced the care burden on the caregivers.

### The Impact of Formal Care Systems on FCP

Something that fundamentally influences FCP is the differences due to existing formal resources, which was alluded to in a few studies but was highlighted in two studies specifically. Pinquart et al. (2003) found national differences in preparing for future care needs at old age, which were explained by variation in beliefs about the usefulness of such planning among the study population (US and Germans). The study noted that the beliefs are a proxy of the participants’ understanding of the stability of the healthcare system of their respective countries and values attached to health-related concerns. For example, Germany has nationwide long-term health care insurance and thus, older adults there have to worry less about making arrangement for care situation in comparison to Americans. However, within each country, there were differences, such as East Germans having fewer concrete plans than West Germans, reflecting the changes the former group went through after the reunification of the country. Also, within the US, participants from Georgia finding formal care services deficient in the state were more likely to find family members as a source of support than those from Utah. Factors that promote and prevent FCP were also investigated by Pinquart and Sörensen (2002b), who came to the conclusion that the older women in their sample coped with the unpredictability in connection to their FCP by either not making plans at all, or by making general plans, which could be adapted once more acute care needs were to arise.

It becomes clear that even though older adults engage in planning behaviours related to FCP, their capacities to make these different kinds of plans are tightly interwoven with each other. For example, someone might not engage in any planning behaviour because they have a strong belief in the stability of the healthcare system in which they live, as Pinquart and colleagues (2003) point out, while others might plan extensively because they lack trust in the stability of their healthcare system.

## Discussion

This scoping review is one of the first studies that synthesises existing research on FCP outside an institutionalized care context and explores how older adults living in the community across different cultural and national contexts prepare for their increasing health, financial and other care needs. Only 12 studies are included in our review which reflects the lacking attention paid to this critical issue of FCP carried out much ahead of any care needs materializing. As stated previously, we excluded studies related to FCP which concentrate on tools that examine care planning (Allen et al., 2018; Sörensen et al., 2017; Sörensen and Pinquart, 2001; Wolff et al., 2021) as well as studies that have explored different aspects of end-of-life care planning within the concept of ACP (Carr & Luth, 2017; Dickinson et al., 2013; Hickman et al., 2023; Mullick et al., 2013). We thus emphasize the need for studies that tackle this problem of relatively limited planning that is done to make support arrangements for old age needs much before actual dependency strikes. Such studies are important not only from the perspective of older persons and their family members, but also from the perspective of the government in light of the shrinking public funding and benefits at old age (Bongaarts, 2004; Bonoli & Palier, 2007; Börsch-Supan & Wilke, 2004; Disney, 2000).

Second, using the included studies, we discussed emergent themes such as future caregiver preferences, living arrangements, financial and health-related planning. Our analysis highlights significant variability in how older adults approach FCP, influenced by factors such as availability of family carers, housing arrangements, cultural values, financial resources, and the formal care systems in which they live. More specifically, a key finding across several studies is the reliance on family members for future care, particularly in societies where filial responsibility is deeply rooted, such as in China (Bai et al., 2022). In contrast, findings from the U.S. and other countries showed more diverse preferences, with some participants opting for formal care services, such as home care or nursing homes. Future living arrangements also emerged as an important theme. Studies like those by Roberto and colleagues (2001) and Sörensen and Pinquart (2000) suggested that wealthier participants were more likely to have concrete plans, such as securing long-term care insurance or places in retirement communities. However, participants from lower-income groups, or those in countries with limited formal care options, exhibited more reluctance to engage in planning. Financial insecurity remains a significant barrier to FCP (Girling & Morgan, 2014; Sörensen and Pinquart, 2000). We have also found that the strength of formal care systems and national policies and the lack thereof significantly influence FCP practices. Our findings highlighted that older adults in countries with robust long-term care insurance systems (e.g., Germany) are more likely to engage in care planning compared to those in countries with weaker formal care infrastructures, such as the U.S. We did not find studies from low and middle-income countries discussing this topic, which calls for the need to study these themes not only from micro to macro level factors but also across different country contexts. Studying FCP contextualized within the socio-economic-cultural-legal settings is critical as care provided within the family is linked by the benefits of the welfare state as well as cultural and legal filial obligations (Daatland et al., 2011; Haberkern & Szydlik, 2010).

In addition to the limited corpus of studies that we could include in our scoping review, it is evident that more research is also necessary on topics such as identifying barriers faced by older adults in planning for their old age (Bai et al., 2022; Delgadillo et al., 2004; Kozlov et al., 2021; Pinquart et al., 2003), and examining how these barriers differ for minorities within a country as well as comparing them with studies from other countries. Emphasis could also be placed on studying specific populations that are expected to have prolonged health related concerns such as persons with chronic illness or those living alone (Kim et al., 2014). At the meso level, there is need to find out more about the role of healthcare professionals and family members in the process of FCP discussions and how the actual progressions towards planning should be done (Girling & Morgan, 2014). In addition to cross sectional qualitative and quantitative studies, there is a need to have studies that employ randomized controlled trials (Orsulic-Jeras et al., 2019) and longitudinal study designs (Sörensen and Pinquart, 2000, 2001) to present the evidence of the highest level possible.

This scoping review has a number of limitations. It is important to note, that only English-language studies, published between January 1, 2000 and December 31, 2023 were included. Also, we only included studies that were found based on the search terms that the research team agreed upon and which were found in the databases Pubmed incl. Medline, CINAHL, PsycINFO, SocINDEX, Web of Science, Scopus, Embase and Google Scholar. It is therefore possible, that studies from different databases, in other languages than English, or those from other terminologies that we did not include in our search terms, were not included in this review. Finally, we excluded studies on older adults already in need of health and long-term care, for example those in institutionalized care setting and studies related to end-of-life decision making, which compose a huge portion of care planning carried out for older persons. Including these topics would have increased the number of studies eligible for this study but would not have contributed any new knowledge than what we already know for topics related to medical decision making, particularly those surrounding the end-of-life.

## Conclusion

The findings from this review make clear that there is very limited research focusing on FCP carried out when one is healthy and living in the community. We also find that there are individual differences based on experiences as well as personal circumstances of the older person when it comes to FCP. That is, they are limited in FCP depending on their financial means, their relationships to family members, their socio-cultural context, the healthcare system, as well as a (potential) lack of formal and informal care resources. However, the studies that were included in this review, focusing on FCP outside of an institutionalized care context, make it apparent that the different planning behaviours displayed are interlinked with each other, meaning that for example, someone might not *need* to display any planning behaviours if they have offspring and life in a socio-cultural context where filial obligations are strongly anchored in society. Similarly, they might face a threatening future if their only offspring has passed away or may not have any offspring or any younger family relative to support them as well as when financial resources are limited. The findings from this review therefore highlight the need to address individual needs with an intersectional perspective considering multiple aspects, which are important to support older adults preparing for the time they might not be able to take care of themselves anymore. Given that cultural norms around family caregiving can either facilitate or hinder formal planning, and financial insecurity continues to be a major barrier to long-term care planning for many, it is essential for governments to consider how interventions and care systems can be adapted to improve the planning process. This would help ensure that care aligns with the values and preferences of older adults, and that the needs of those from lower socioeconomic backgrounds are addressed.

## Supplemental Material

Supplemental Material - Future Care Planning for and by Older Adults Living in the Community: A Scoping Review Supplemental Material for Future Care Planning for and by Older Adults Living in the Community: A Scoping Review by Nathalie Bettina Neeser, Xin Feng, Wei Yang, Ruru Ping, Xue Bai, and Tenzin Wangmo in Research on Aging

## Data Availability

The protocol for this scoping review was registered in Open Science Framework (OSF) and can be accessed online (https://osf.io/r3jpc/). All full searches are available in the supplemental file.
