# Supplementary material for: Future Care Planning for and by Older Adults Living in the Community: A Scoping Review
Source: Res Aging. 2025 Jun 8;48(1):82–95. doi: 10.1177/01640275251348582 (PMC12559365; doi:10.1177/01640275251348582)
Supplement: Supplemental Material - Future Care Planning for and by Older Adults Living in the Community: A Scoping Review [file sj-pdf-1-roa-10.1177_01640275251348582.pdf]

Supplementary Material

Table A. Full search strategy for Embase

**Population I, Population II & Context**	('older adult'/exp OR 'older adult' OR 'older adults'/exp OR 'older adults' OR 'aging'/exp OR 'aging' OR 'aged'/exp OR 'aged' OR 'older people'/exp OR 'older people' OR 'senior resident'/exp OR 'senior resident' OR 'geriatric'/exp OR 'geriatric' OR 'geriatrics'/exp OR 'geriatrics' OR 'gerontology'/exp OR 'gerontology' OR 'older adult*':ab,ti OR 'ag*ng':ab,ti OR 'elder*':ab,ti OR 'aged person*':ab,ti OR 'older person*':ab,ti OR 'elder* people':ab,ti OR 'older parent*':ab,ti OR 'older people':ab,ti OR 'elder* person*':ab,ti OR 'senior*':ab,ti OR 'geriatr*':ab,ti OR 'gerontolo*':ab,ti) **AND** ('informal caregiver'/exp OR 'informal caregiver' OR 'caregiver'/exp OR 'caregiver' OR 'family caregiving'/exp OR 'family caregiving' OR 'adult child'/exp OR 'adult child' OR 'informal caregiv*':ab,ti OR 'unpaid caregiv*':ab,ti OR 'famil* caregiv*':ab,ti OR 'caregiv*':ab,ti OR 'carer*':ab,ti OR 'informal carer*':ab,ti OR 'unpaid carer*':ab,ti OR 'famil* carer*':ab,ti OR 'adult child*':ab,ti OR 'adult daughter*':ab,ti OR 'adult son*':ab,ti) **AND** ('care* plan*':ab,ti OR 'care* prepar*':ab,ti OR 'care* discussion*':ab,ti OR 'social care plan*':ab,ti OR 'long-term care plan':ab,ti OR 'anticipatory care planning':ab,ti)
**With the additionally applied filters: "time" (2000-2023), "human", "language" (English) and "publication type" (Article, Article in press).**	

Table B. Full search strategy for PubMed incl. Medline

**Population I, Population II & Context**	((("Aging"[Mesh]) OR ("Aged"[Mesh]) OR ("Healthy Aging"[Mesh]) OR ("Frail Elderly"[Mesh]) OR ("Geriatrics"[Mesh]) OR ("older adult*"[Title/Abstract]) OR ("aging"[Title/Abstract]) OR ("ageing"[Title/Abstract]) OR ("elder*"[Title/Abstract]) OR ("aged person*"[Title/Abstract]) OR ("older person*"[Title/Abstract]) OR ("elder people"[Title/Abstract]) OR ("elderly people"[Title/Abstract]) OR ("older parent*"[Title/Abstract]) OR ("older people"[Title/Abstract]) OR ("elder person*"[Title/Abstract]) OR ("elderly person*"[Title/Abstract]) OR ("senior*"[Title/Abstract]) OR ("geriatr*"[Title/Abstract]) OR ("gerontolo*"[Title/Abstract])) **AND** (("Caregivers"[Mesh]) OR ("Household Work"[Mesh]) OR ("Adult Children"[Mesh]) OR ("informal caregiv*"[Title/Abstract]) OR ("unpaid caregiv*"[Title/Abstract]) OR ("family caregiv*"[Title/Abstract]) OR ("familiar caregiv*"[Title/Abstract]) OR ("caregiv*"[Title/Abstract]) OR ("carer*"[Title/Abstract]) OR ("informal carer*"[Title/Abstract]) OR ("unpaid carer*"[Title/Abstract]) OR ("family carer*"[Title/Abstract]) OR ("adult child*"[Title/Abstract]) OR ("adult daughter*"[Title/Abstract]) OR ("adult son"[Title/Abstract]) OR ("adult sons"[Title/Abstract]))) **AND** (("Housing for the Elderly"[Mesh]) OR ("care plan*"[Title/Abstract]) OR ("caring plan*"[Title/Abstract]) OR ("caregiving plan*"[Title/Abstract]) OR ("care prepar*"[Title/Abstract]) OR ("caregiving prepar*"[Title/Abstract]) OR ("care discussion*"[Title/Abstract]) OR ("social care plan*"[Title/Abstract]) OR ("long-term care plan"[Title/Abstract]) OR ("anticipatory care planning"[Title/Abstract]))
**With the additionally applied filters: "time" (2000-2023), "human" and "language" (English).**	

Table C. Full search strategy for CINAHL

**Population I, Population II & Context**	(TI ((“older adult*”) OR (“aging”) OR (“ageing”) OR (“elder*”) OR (“aged person*”) OR (“older person*”) OR (“elder people”) OR (“elderly people”) OR (“older parent*”) OR (“older people”) OR (“elder person*”) OR (“elderly person*”) OR (“senior*”) OR (“geriatr*”) OR (“gerontolo*”)) OR AB ((“older adult*”) OR (“aging”) OR (“ageing”) OR (“elder*”) OR (“aged person*”) OR (“older person*”) OR (“elder people”) OR (“elderly people*”) OR (“older parent*”) OR (“older people”) OR (“elder person*”) OR (“elderly person*”) OR (“senior*”) OR (“geriatr*”) OR (“gerontolo*”))) **AND** (TI ((“informal caregiv*”) OR (“unpaid caregiv*”) OR (“family caregiv*”) OR (“familiar caregiv*”) OR (“caregiv*”) OR (“carer*”) OR (“informal carer*”) OR (“unpaid carer*”) OR (“family carer*”) OR (“familiar carer*”) OR (“adult child*”) OR (“adult daughter*”) OR (“adult son*”)) OR AB ((“informal caregiv*”) OR (“unpaid caregiv*”) OR (“family caregiv*”) OR (“familiar caregiv*”) OR (“caregiv*”) OR (“carer*”) OR (“informal carer*”) OR (“unpaid carer*”) OR (“family carer*”) OR (“familiar carer*”) OR (“adult child*”) OR (“adult daughter*”) OR (“adult son*”))) **AND** (TI ((“care plan*”) OR (“caring plan*”) OR (“caregiving plan*”) OR (“caretaking plan*”) OR (“care prepar*”) OR (“caring prepar*”) OR (“caregiving prepar*”) OR (caretaking prepar*”) OR (“care discussion*”) OR (“caring discussion*”) OR (“caregiving discussion*”) OR (“caretaking discussion*”) OR (“social care plan*”) OR (“long-term care plan”)) OR AB ((“care plan*”) OR (“caring plan*”) OR (“caregiving plan*”) OR (“caretaking plan*”) OR (“care prepar*”) OR (“caring prepar*”)OR (“caregiving prepar*”) OR (caretaking prepar*”) OR (“care discussion*”) OR (“caring discussion*”) OR (“caregiving discussion*”) OR (“caretaking discussion*”) OR (“social care plan*”) OR (“long-term care plan”) OR (“anticipatory care planning”)))
**With the additionally applied filters: “time” (2000-2023) and “language” (English).**	

Table D. Full search strategy for PsycInfo

**Population I, Population II & Context**	(("older adult*" or "aging" or "ageing" or "elder*" or "aged person*" or "older person*" or "elder* people" or "older parent*" or "older people" or "elder* person*" or "senior*" or "geriatr*" or "gerontolo*").ti. or ("older adult*" or "aging" or "ageing" or "elder*" or "aged person*" or "older person*" or "elder* people" or "older parent*" or "older people" or "elder* person*" or "senior*" or "geriatr*" or "gerontolo*").ab.) **and** (("informal caregiv*" or "unpaid caregiv*" or "famil* caregiv*" or "caregiv*" or "carer*" or "informal carer*" or "unpaid carer*" or "famil* carer*" or "adult child*" or "adult daughter*" or "adult son*").ti. or ("informal caregiv*" or "unpaid caregiv*" or "famil* caregiv*" or "caregiv*" or "carer*" or "informal carer*" or "unpaid carer*" or "famil* carer*" or "adult child*" or "adult daughter*" or "adult son*").ab.) **and** (("care* plan*" or "care* prepar*" or "care* discussion*" or "social care plan*" or "long-term care plan" or "anticipatory care planning").ti. or ("care* plan*" or "care* prepar*" or "care* discussion*" or "social care plan*" or "long-term care plan" or "anticipatory care planning").ab.))
**With the additionally applied filters: “time” (2000-2023), “human” and “language” (English).**	

Table E. Full search strategy for SocINDEX

**Population I**	(DE "OLDER people") OR (DE "AGING") OR (DE "AGING parents") OR (DE "GERONTOLOGY") OR (DE "GERIATRICS") OR TI (("older adult*") OR ("ag*ng") OR ("elder*") OR ("aged person*") OR ("older person*") OR ("elder* people") OR ("older parent*") OR ("older people") OR ("elder* person*") OR ("senior*") OR ("geriatr*") OR ("gerontolo*")) OR AB (("older adult*") OR ("ag*ng") OR ("elder*") OR ("aged person*") OR ("older person*") OR ("elder* people") OR ("older parent*") OR ("older people") OR ("elder* person*") OR ("senior*") OR ("geriatr*") OR ("gerontolo*")) OR KW (("older adult*") OR ("ag*ng") OR ("elder*") OR ("aged person*") OR ("older person*") OR ("elder* people") OR ("older parent*") OR ("older people") OR ("elder* person*") OR ("senior*") OR ("geriatr*") OR ("gerontolo*"))
**Population II**	(DE "ADULT children of aging parents") OR (DE "FAMILY relationships of adult children of aging parents") OR (DE "CHILD caregivers") OR (DE "CAREGIVERS") OR (DE "CARING") OR (DE "ADULT children") OR TI (("informal caregiv*") OR ("unpaid caregiv*") OR ("famil* caregiv*") OR ("caregiv*") OR ("carer*") OR ("informal carer*") OR ("unpaid carer*") OR ("famil* carer*") OR ("adult child*") OR ("adult daughter*") OR ("adult son*")) OR AB (("informal caregiv*") OR ("unpaid caregiv*") OR ("famil* caregiv*") OR ("caregiv*") OR ("carer*") OR ("informal carer*") OR ("unpaid carer*") OR ("famil* carer*") OR ("adult child*") OR ("adult daughter*") OR ("adult son*")) OR KW (("informal caregiv*") OR ("unpaid caregiv*") OR ("famil* caregiv*") OR ("caregiv*") OR ("carer*") OR ("informal carer*") OR ("unpaid carer*") OR ("famil* carer*") OR ("adult child*") OR ("adult daughter*") OR ("adult son*"))
**Context**	TI (("care* plan*") OR ("care* prepar*") OR ("care* discussion*") OR ("social care plan*") OR ("long-term care plan") OR ("anticipatory care planning")) OR AB (("care* plan*") OR ("care* prepar*") OR ("care* discussion*") OR ("social care plan*") OR ("long-term care plan") OR ("anticipatory care planning")) OR KW (("care* plan*") OR ("care* prepar*") OR ("care* discussion*") OR ("social care plan*") OR ("long-term care plan") OR ("anticipatory care planning"))
**With the additionally applied filters: "time" (2000-2023) and "language" (English).**	

Table F. Full search strategy for Web of Science

**Population I, Population II & Context**	((AB=(("informal caregiv*") OR ("unpaid caregiv*") OR ("famil* caregiv*") OR ("caregiv*") OR ("carer*") OR ("informal carer*") OR ("unpaid carer*") OR ("famil* carer*") OR ("adult child*") OR ("adult daughter*") OR ("adult son*"))) **AND** AB=(("older adult*") OR ("aging") OR ("ageing") OR ("elder*") OR ("aged person*") OR ("older person*") OR ("elder* people") OR ("older parent*") OR ("older people") OR ("elder* person*") OR ("senior*") OR ("geriatr*") OR ("gerontolo*"))) **AND** AB=(("care* plan*") OR ("care* prepar*") OR ("care* discussion*") OR ("social care plan*") OR ("long-term care plan") OR ("anticipatory care planning"))
**With the additionally applied filters: "time" (2000-2023) and "language" (English).**	

Table G. Full search strategy for Scopus

**Population I, Population II & Context**	(TITLE-ABS-KEY (("older adult*") OR ("aging") OR ("ageing") OR ("elder*") OR ("aged person*") OR ("older person*") OR ("elder* people") OR ("older parent*") OR ("older people") OR ("elder* person*") OR ("senior*") OR ("geriatr*") OR ("gerontolo*")) **AND** TITLE-ABS-KEY (("informal caregiv*") OR ("unpaid caregiv*") OR ("famil* caregiv*") OR ("caregiv*") OR ("carer*") OR ("informal carer*") OR ("unpaid carer*") OR ("famil* carer*") OR ("adult child*") OR ("adult daughter*") OR ("adult son*")) **AND** TITLE-ABS-KEY (("care* plan*") OR ("care* prepar*") OR ("care* discussion*") OR ("social care plan*") OR ("long-term care plan")))
**With the additionally applied filters: "time" (2000-2023), "human" and "language" (English).**	

Table H. Full search strategy for Google Scholar

**Population I, Population II & Context**	(((“older adult*”) OR (“aging”) OR (“ageing”) OR (“elder*”) OR (“aged person*”) OR (“older person*”) OR (“elder* people”) OR (“older parent*”) OR (“older people”) OR (“elder* person*”) OR (“senior*”) OR (“geriatr*”) OR (“gerontolo*”)) **AND** ((“informal caregiv*”) OR (“unpaid caregiv*”) OR (“famil* caregiv*”) OR (“caregiv*”) OR (“carer*”) OR (“informal carer*”) OR (“unpaid carer*”) OR (“famil* carer*”) OR (“adult child*”) OR (“adult daughter*”) OR (“adult son*”)) **AND** ((“care* plan*”) OR (“care* prepar*”) OR (“care* discussion*”) OR (“social care plan*”) OR (“long-term care plan”) OR (“anticipatory care planning”)))
**With the additionally applied filter: “time” (2000-2023).**	
